# Evidence of wet-dry cycles and mega-droughts in the Eemian climate of southeast Australia

**DOI:** 10.1038/s41598-020-75071-z

**Published:** 2020-10-22

**Authors:** Hamish McGowan, Micheline Campbell, John Nikolaus Callow, Andrew Lowry, Henri Wong

**Affiliations:** 1 Atmospheric Observations Research Group, The University of Queensland, Brisbane, Australia; 2 School of Agriculture and Environment, The University of Western Australia, Perth, Australia; 3 Australian Nuclear Science and Technology Organisation, Lucas Heights, Sydney, Australia

**Keywords:** Climate sciences, Environmental social sciences

## Abstract

Understanding past climate variability is critical to informing debate of likely impacts of global warming on weather and climate, and water resources. Here we present a near annual resolution reconstruction of climate developed from a speleothem that spans the Eemian [Marine Isotope Stage 5e (MIS 5e)] from 117,500 to 123,500 years BP—the most recent period in the Earth’s history when temperatures were similar to those of today. Using ^25^ Mg, ^88^Sr, and ^137^Ba as proxies, we show the first indication of solar and teleconnection cyclic forcing of Eemian climate in southeast Australia, a region at present often affected by severe drought and bushfires. We find evidence for multi-centennial dry periods interpreted as mega-droughts, and highlight the importance of understanding the causes of these in the context of a rapidly warming world, where temperatures are now, or projected to exceed those of the Eemian.

## Introduction

The Eemian or peak of the Last Interglacial [Marine Isotope Stage 5e (MIS 5e)*;* ca. 129,000–116,000 years Before Present (BP)] is the most recent geologic period when global temperatures were similar to present, but in response to orbital forcing rather than greenhouse gas loading of the atmosphere. While this makes the Eemian an imperfect analogue for near-future climate due to anthropogenic global warming, the latitudinal temperature distribution was similar to the present^1–4^. Eemian global mean temperature was 0—2 °C warmer than present, mean global sea surface temperatures (SSTs) were indistinguishable from current SSTs^5^, though sea level was around 6–9 m higher from meltwater inflows from the Greenland and Antarctic ice sheets. Therefore, understanding the climate of the Eemian may provide valuable insight to future climate and its variability.

While often thought of as a period of relative climate stability, climate variability during the Eemian was likely greater than in the Holocene. This has been attributed to meltwater outflows disturbing the Atlantic meridional overturning circulation (AMOC)^6,7^ and a general global cooling trend toward glacial inception^8^. The resulting changes in sea surface salinity and temperature are believed to have driven regional changes in atmospheric circulation leading to periods of widespread aridity across Europe^6^, and onset of abrupt cold periods as SSTs cooled by several degrees Celsius^6,9^.

Our understanding of Southern Hemisphere Eemian climate is limited as proxy records are rare. The climate of Australia is globally relevant, as it responds to key global teleconnection forcing, including El Niño Southern Oscillation (ENSO), Pacific Decadal Oscillation (PDO) and the Indian Ocean Dipole (IOD). It is also relevant as the severity of recent droughts and bushfires have been linked to increasing temperatures caused by anthropogenic greenhouse gas emissions^10–13^, thereby stimulating interest in understanding past warm climate conditions such as during MIS 5e.

Numerical modelling studies suggest reduced seasonal contrast in MIS 5e temperatures across Australia with warmer winter temperatures, most notably in the north and northwest of the continent by + 3 °C to + 5 °C^14^. Austral summer (December–February) temperatures are believed to have been cooler by − 1 °C to − 2 °C with modelling studies suggesting that the Australian monsoon was weaker and drier than present with January—April precipitation anomalies of > − 3 mm per day^14,15^. A weaker MIS 5e monsoon aligns with evidence of a variable Lake Eyre hydrology with temporally isolated small inflow events^16^. These were likely caused by irregular southward penetration of the Intertropical Convergence Zone (ITCZ) and decaying tropical cyclone(s) over the headwaters of the Lake Eyre Basin—conditions similar to those of today that deliver inflows to Lake Eyre.

Winter temperatures in southern Australia during MIS 5e are believed to have been warmer than present, reflecting warmer SSTs in the Southern Ocean. This would have reduced the meridional temperature gradient between the sub-tropics and mid/high latitudes with less frequent precipitation bearing cold fronts and extra-tropical depressions at a time concurrent with strengthening of interannual variability of SSTs in the eastern tropical Pacific associated with ENSO^17^. Slightly cooler MIS 5e SSTs to the northwest of Australia relative to present^5,18^ may have contributed further to reduced rainfall over southeast Australia similar to the impact of present day positive IOD events^19^. Combined with changes to AMOC and associated SSTs, which have been shown under present day conditions to teleconnect to the Pacific Ocean^20^, the climate of MIS 5e in Australia is likely to have been variable, but in general drier than present. In particular, in southeast Australia where under the current climate positive IOD and ENSO result in reduced rainfall^20–22^. However, a dearth of high temporal resolution paleoclimate records from MIS 5e have until present not been available to test this thesis and the possible links to climate state forcings such as teleconnections and solar variability.

**Figure 1:**
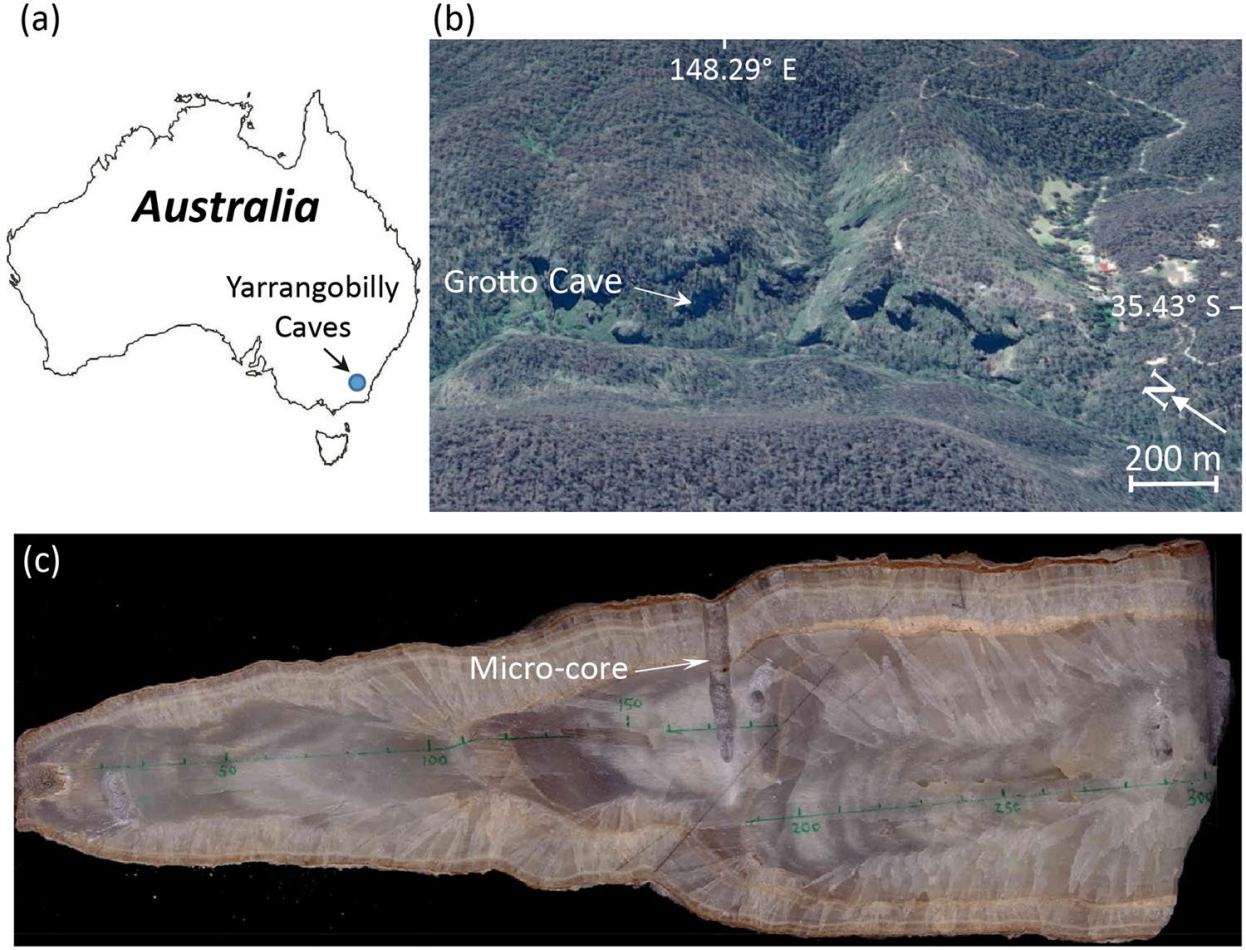
Location map of the Yarrangobilly Caves **(a)** and aerial oblique perspective of the entrance to the Grotto Cave created using Google Earth Pro 7.3 (Google Earth, earth.google.com/web/) **(b)**. 300 mm long cross-section of stalagmite GC001 from which samples were extracted for uranium series dating (see Table S1) and geochemical analysis **(c)**. The small core perpendicular to the growth axis is the result of in-situ sampling of GC001 for age determination prior to removal from the Grotto Cave **(c)**.

In Australia, speleothems offer the greatest potential to develop high temporal resolution terrestrial paleo-environmental records. Stalagmite GC001 was removed from a small chamber approximately 60 m into the Grotto Cave, Yarrangobilly Caves, New South Wales, Australia [35.43° S; 148.29° E; 935 m Australian Height Datum (AHD)] in 2012 (Fig. 1) under New South Wales Parks and Wildlife Scientific licence SL100538. The caves are located at the northern end of the Snowy Mountains, at an elevation where the water balance changes from an energy (demand) to supply (precipitation) limited system^23,24^. The caves were formed through karstification of the Yarrangobilly Limestone, a massive Silurian limestone formation with an extent of ~ 1.4 km by 14 km and a maximum depth of ~ 450 m^25^. Past work has concluded that reduced rainfall invokes prior calcite precipitation (PCP) at Yarrangobilly and hence up (down) trend in Mg/Ca ratios implies increased (decreased) drip water contact time under drier (wetter) conditions^26,27^.

The present day climate of the Yarrangobilly area is influenced by the northern extension of the mid-latitude westerly winds. It experiences cool to cold montane temperatures through winter, while in summer warm to hot and dry conditions dominate under the influence of the subtropical ridge. Major Southern Hemisphere ocean–atmosphere teleconnections including the ENSO, Southern Annular Mode (SAM), PDO, and IOD all affect the regional climate state^28–32^. Rain bearing weather systems that affect Yarrangobilly Caves may originate from the tropics and the southern mid-latitudes^33,34^. Mean annual precipitation is approximately 1147 mm, with a mean annual temperature of 12 °C^35,36^. The most effective precipitation occurs during the cool, wet winter^36,37^ and while snow may fall, the site is below the seasonal snowline and no snowpack remains through winter. Temperature logging from where GC001 was collected in the Grotto Cave found mean internal cave temperature was 8.8 °C during the study (August 2014 to February 2015) .

## Results

**Figure 2:**
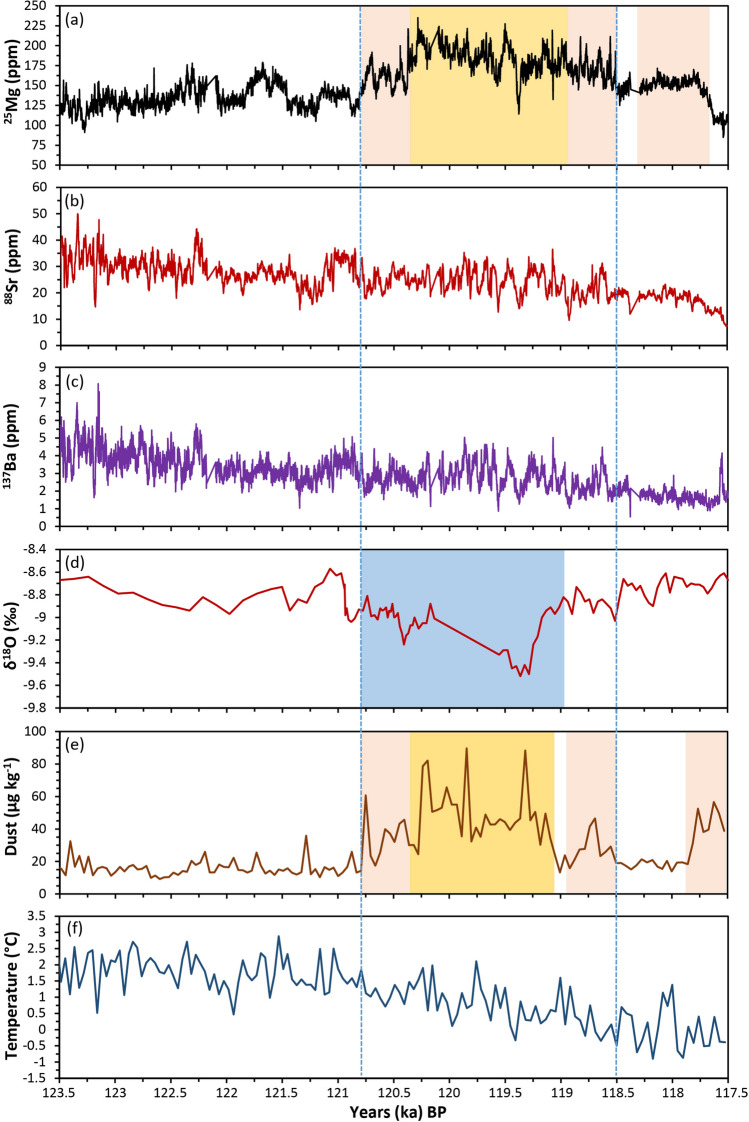
Time series of Mg, Sr and Ba concentrations from GC001 **(a–c)**; δ^18^O from a speleothem collected at Whiterock Cave (4^◦^6′ N, 114^◦^53′ E), northern Borneo^38^ **(d)**; dust concentrations from Dome C (75°06′ S, 123°21′ E), Antarctic^41^ **(e)**, and temperature departure from the average of the past 1000 years Dome C, (75°06′ S, 123°21′ E), Antarctic^42^ **(f)**. Pink shading indicates periods of intermediate Mg concentrations and dust flux **(a,e)**; yellow shading highest Mg concentrations and dust flux **(a,e)** and blue shading **(d)** corresponding period of depleted δ^18^O indicating wetter conditions in Western Pacific. Vertical dashed blue lines constrain the period interpreted as a mega-drought(s).

The Mg time series for GC001 (Fig. 2a) shows clear cycles of higher/lower concentrations with a marked increase occurring around 120,800 years BP. This period of elevated Mg concentrations prevailed until around 118,500 years BP. It then remained mostly stable until a sharp decrease at approximately 117,850 years BP. This period of higher Mg concentrations (drier) in GC001 aligns with a period of increased depletion of δ^18^O in a speleothem from northern Borneo^38^ indicating wetter conditions there (Fig. 2d), indicative of a more northerly position of the ITCZ. It also correlates with periods of increased dust deposition recorded at Dome C, Antarctica (Fig. 2e). Australia is a known source of interglacial dust to the Antarctic^39,40^. Accordingly, we infer drier conditions in southeast Australia as indicated by higher Mg concentrations from 120,800 to 118,500 years BP in GC001 sustained regional mega-drought(s) across this period with associated wind erosion contributing to increased dust flux at Dome C, Antarctica such as between 119,050 to 120,300 years BP (Fig. 2e). Slightly higher dust flux values from 117,500 to 117,800 years BP (Fig. 2e) may have also originated from southeast Australia but do not correlate with elevated Mg concentrations in GC001 (Fig. 2a), or any notable signal in the δ^18^O record from northern Borneo (Fig. 2d).

The Sr time series (Fig. 2b) displays some periods of higher/lower concentrations that are concurrent with variability in the Mg record (Fig. 2a), such as around 121,000 years BP. However, unlike the Mg record, the Sr time series exhibits an overall trend of decreasing concentrations with the period between approximately 120,800 years to 118,500 years BP remaining relatively constant at approximately 25 ppm, before decreasing more quickly. This is similar to the Ba concentrations time series (Fig. 2c) which is strongly correlated with Sr (R^2^ = 0.80, n = 3980) reflecting a reduced growth rate (less drip water) under the drier conditions known to have minimal impact on Mg concentrations^43^. Ba concentrations do display a more consistent decreasing trend that flattens between 120,800 and 119,100 years BP before decreasing further after 118,500 years BP. While exogenic factors such as dust may have affected the Ba record, this is not distinguishable in Fig. 2c when compared to records such that shown in Fig. 2e. The decreasing trends in the Sr and Ba concentrations correlate with the MIS 5e temperature anomaly reconstruction for Dome C, Antarctic (Fig. 2f) and MIS 5e SST reconstructions from northwest of Australia and east of New Zealand^44,45^. Accordingly, the Mg, Sr and Ba concentrations in GC001 collectively record the onset of a prolonged drier period from around 120,750 years BP to 118,500 years BP (Fig. 2) that was associated with increased atmospheric dust and a cooling in atmospheric and ocean temperatures from MIS 5e maximums.

**Figure 3:**
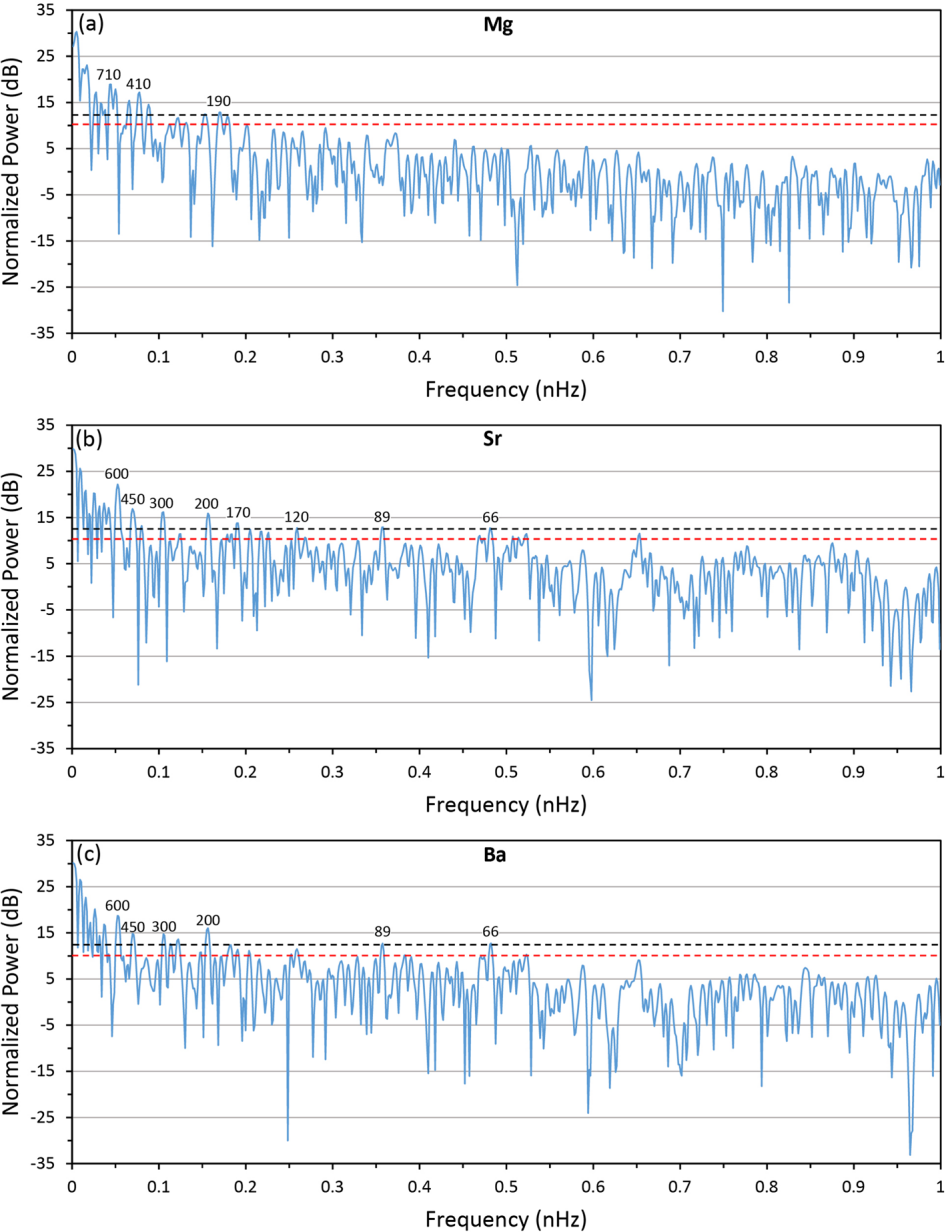
Periodograms of Mg, Sr and Ba with 0.9 (red dashed line) and 1.0 probability thresholds shown. Selected dominant periods (annotated) are rounded to the nearest decade.

Normalised Lomb-Scargle periodograms for Mg, Sr and Ba were calculated to test for the influence of teleconnection and/or solar cycle forcing on the MIS 5e climate of southeast Australia (Fig. 3). The periodograms show common peaks in spectral density around 200 yrs which align with the de Vries solar cycle (~ 205 years)^46^. Spectral peaks are also found from 1147 yrs (Mg) to 1268 yrs (Sr) and are within the range of the ~ 1000 year Eddy solar cycle^47,48^. The Sr and Ba records have peaks at ~ 66.8 and 88.6 years, which match with the PDO and Gleissberg solar cycle respectively and display coherent spectral peaks at 301.3 years, 454.8 years, 602.7 years and at 3443.8 years. The 301.3 years and 454.8 years cycles are close to the 300 year and 470 year cycles found in sediments from Jeju Island, South Korea^49^. These have been attributed to solar forcing along with the 3443.8 year cycle, which is aligned with the 3300 year Holocene cycles in the δ^18^O record from GISP2^49,50^. The Mg record displays a strong 709 year cycle that may be a harmonic of the 1400 year cycle found in glacial and interglacial periods in Mg/Ca derived SST records from the South China Sea^51^. Accordingly, the geochemistry of GC001 displays cyclic variability indicative of climate variability forced by both teleconnections and solar cycles recorded globally in geologic archives.

**Figure 4:**
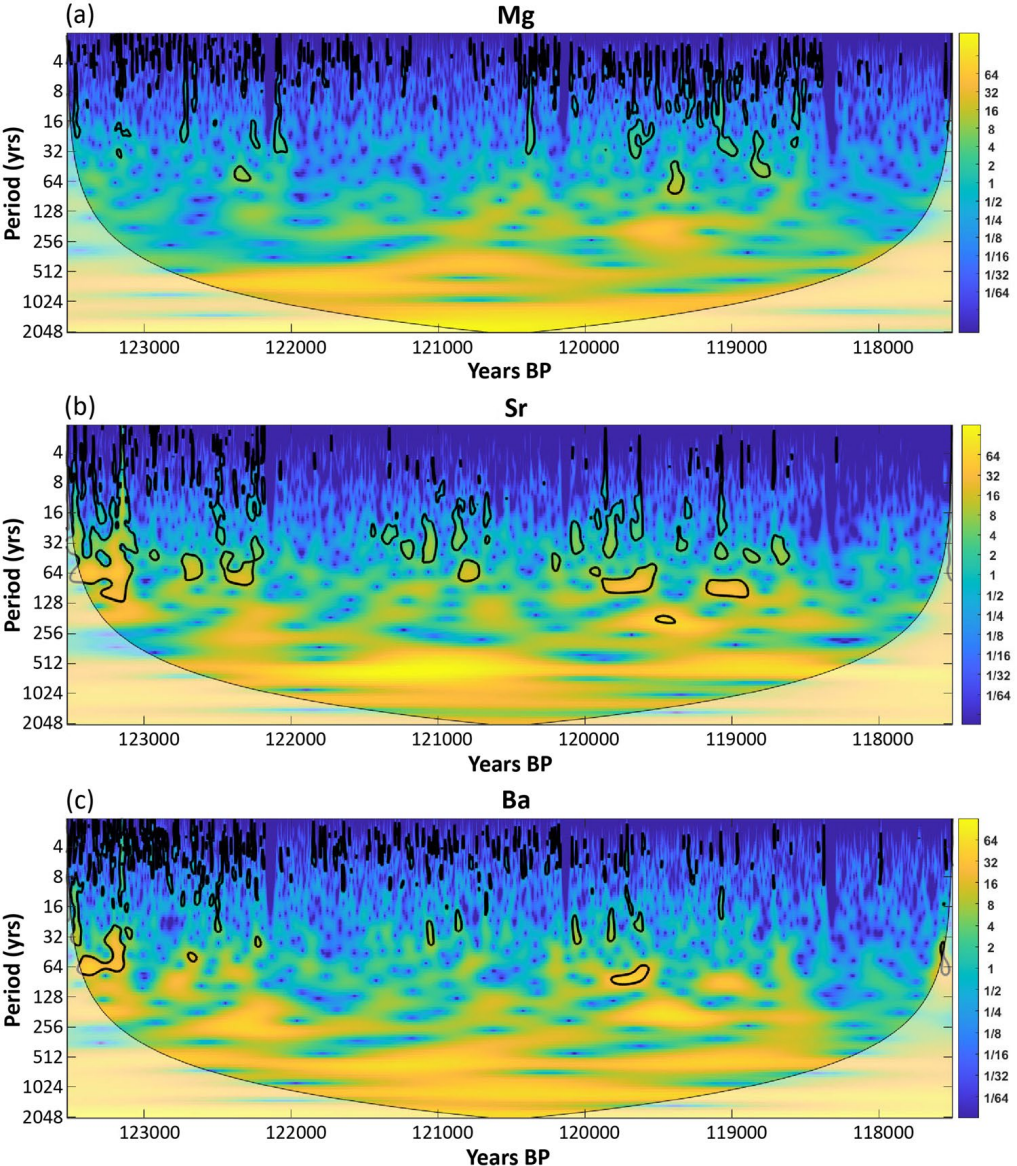
Wavelet plots of Mg **(a)**, Sr **(b)**, and Ba **(c)**. The thick black lines represent 95% confidence levels. The lightly shaded region toward the bottom is the Cone of Influence and values in the light region should be considered with caution.

Wavelet transforms for Mg, Sr and Ba presented in Fig. 4 show regions of significant periodicity outlined in black (95% confidence level). All three elements display significant 4 to 8 year cycles that we interpret as ENSO and around 20 to 30 yrs, and 50 to 65 yrs, which are indicative of the PDO. While caution is required in attributing shorter-duration cycles given laser ablation sampling resolution, the Mg record shows clear gaps in ENSO (4–8 year) cycles from approximately 121,500 years BP to around 120,500 years BP. These coincide with a change in Mg concentrations from ~ 140 to ~ 200 ppm indicating a transition to drier conditions, while the period from 118,500 to 117,500 years BP overlaps with the return to Mg concentrations ~ 140 ppm suggesting increased moisture availability. PDO like cycles are most common from around 119,800 years BP to 118,500 years BP.

In the Sr wavelet transform diagram (Fig. 4b), the ENSO signals are most frequent from the start of the record to about 122,100 years BP and are concurrent with the occurrence of the multi-decadal PDO like cycle. This longer decadal cyclicity is also evident from 121,500 years BP to 118,500 years BP with evidence of a separate 20 to 30 year cycle and longer 55 to 75 year cycle. The Ba wavelet plot (Fig. 4c) shows also a dominant short duration ENSO signal in the early part of the record that progressively becomes less frequent. The PDO period cycle is not strong and only occurs above the 95% confidence level occasionally from 123,500 years BP to around 122,200 years BP and again from around 121,000 years BP to 119,500 years BP.

## Discussion

Understanding the causes and frequency of climate variability is essential to inform prediction of future climate. Proxy of climate variability dating from MIS 5e, the most recent warm period with temperatures similar to the present, offer potential to develop this understanding. The Mg MIS 5e record from stalagmite GC001 shows multi-centennial periods of increased Mg concentration indicating drier climatic conditions in southeast Australia, notably from 118,500 to 120,750 years BP. This period coincides with increased dust flux recorded in Antarctic ice (Fig. 2e). The Sr and Ba concentrations show evidence also of this climate variability (Fig. 2b,c) with trends of decreasing concentrations correlated with cooling air temperatures and SSTs. Collectively, the Mg, Sr and Ba records from GC001 with supporting regional paleoclimate records suggest that southeast Australia during MIS 5e experienced multi-centennial periods of reduced precipitation, temperature variability and increased atmospheric dust. These conditions such as from 118,900 to 120,400 years BP are indicative of mega-droughts (Fig. 2). Superimposed on these multi-centennial periods of drier climatic conditions are higher frequency interannual to interdecadal teleconnections and solar forced cycles of further variability (Fig. 4).

A drier climate during MIS 5e such as from 118,900 to 120,400 years BP would align with results from numerical modelling which found a generally weaker summer monsoon and warmer winter conditions^15^. A weaker monsoon aided by cooler SSTs northwest of Australia at this time^5,18^ would lessen moisture transport into southeast Australia via meridional conveyors such as northwest cloud bands, while warmer temperatures during winter would have increased evaporation, intensifying dry conditions that spanned centuries as evident in our record. Numerical modelling of future climate at + 1.5 °C indicates that the Australian monsoon may weaken, resulting in an increase in the frequency of dry days^52–54^. Accordingly, the convergence of our paleoclimate record from stalagmite GC001 and published numerical modelling strongly suggest that the climate of southeast Australia will likely become drier throughout the twenty-first century with increased risk of multi-centennial duration dry periods widely referred to as mega-droughts.

The Mg, Sr and Ba records from stalagmite GC001 all display cycles with periodicity of well documented solar and teleconnection forcings of climate. Dominant solar cycles including the Gleissberg solar cycle (88 years), de Vries solar cycle (~ 205 years) and possible Eddy solar cycle (~ 1000 years) are evident in the geochemistry of GC001. Increases (decreases) in total solar irradiance (TSI) through such cycles result in direct heating (cooling) of the troposphere and Earth’s surface. Indirectly, changes in TSI influence galactic cosmic ray (GCR) flux affecting cloud microphysics and cloud cover^55^. Variation in TSI also causes change in ultraviolet (UV) radiation flux, which for a 0.1% increase in TSI, increases by 4 to 8%^56^. Concurrently this will increase ozone production in the mid and upper stratosphere through the photolysis of oxygen. Corresponding increases in UV absorption by stratospheric ozone leads to heating and change in the thermal stratification of the atmosphere.

Numerical modelling has shown that enhanced UV heating of the atmosphere corresponding to TSI maxima results in a stratospheric zonal wind anomaly that is most pronounced in the Southern Hemisphere during mid to late winter^57^. Dynamical links between the stratosphere and troposphere caused by such UV forcing lead to change in atmospheric circulation, including jet-stream behaviour, and therefore tropospheric synoptic circulation and ocean dynamics^58^. We therefore postulate that periods of increased TSI contribute to drier conditions (increased Mg in GC001) over southeast Australia in response to increased zonal (westerly) flow and a corresponding more southern track of mid-latitude winter storm systems. This occurs in response to a stronger thermal wind component between the tropics/subtropics and the mid to high latitudes causing a response in atmospheric circulation similar to a more positive SAM. The associated decline in precipitation is recorded clearly in stalagmite GC001 by elevated Mg concentrations and stable to slight decreases in Sr and Ba concentrations due to reduced speleothem growth, i.e. reduced drip rate^43^ during MIS 5e.

GC001 records 4 to 8 years ENSO-like cycles along with multi-decadal cyclicity indicating PDO type influences—two teleconnections which have been shown to have the greatest impact on the modern climate and in particular rainfall in southeast Australia^21,28,59^. It is therefore reasonable to suggest that these Pacific Ocean teleconnections have been robust and long-lived drivers of interannual and multi-decadal hydroclimate variability in southeast Australia with warm/cool ENSO and PDO phases causing dry/wet periods.

Evidence of ENSO cycles in GC001 become no longer statistically significant from around 118,200 yrs BP. This is about 500 years before rapid decline in Mg concentrations, which suggests reduction in drip water residence times and onset of a wetter climate. This change in Mg and to a lesser amount in Sr and Ba correspond to a rapid increase in sea level of around 5 to 6 m at 118,100  ± 1400 years BP caused by ice sheet melt^60^. We suggest that this dramatic rise in sea level possibly disrupted ENSO and PDO teleconnections and their impact on the climate of southeast Australia at this time.

Collectively, the MIS 5e climate record developed from GC001 indicates that southeast Australia will likely continue to experience interannual to interdecadal wet–dry cycles driven by teleconnections and solar variability at least until current global warming exceeds Eemian temperatures, possibly within the next decade. However, our record also shows that in a warm interglacial climate such as today or near future, there is risk of multi-centennial periods of less effective precipitation (mega-droughts), initiated by natural variability. Should such prolonged periods of drier conditions occur again, then they may be reinforced by anthropogenic global warming, thereby increasing their severity. As a result, we stress the need for further research into the Eemian climate of Australia and the Southern Hemisphere to resolve the causes of prolonged dry periods during MIS 5e and to determine their spatial impacts. This will allow new insights to our future climate and the risks it may bring such as drought and associated bushfires.

## Methods

### Sample preparation

Six 50 mm × 8 mm × 10 mm sections sampled from the growth axis of stalagmite GC001 (Fig. 1c) were polished to 1 μm and cleaned with Milli-QTM water in an ultrasonic bath, then dried at 60 °C. ^43^Ca, ^88^Sr, ^25^ Mg and ^137^Ba data were obtained by Laser-Ablation Inductively-Coupled Plasma Mass-Spectrometry at the Australian Nuclear Science and Technology Organisation using a Resonetics M50 193 nm Excimer laser ablation system coupled to a Varian 820–ICP MS. A rectangular laser spot (340 μm × 50 μm) and a laser pulse frequency of 10 Hz was applied with the laser and the narrow side of the rectangle aligned perpendicular to the growth axis, to ensure maximum sampling within the same growth band. Ablation paths were cleaned by laser at a rate of 150 μm s^−1^ prior to analysis at 30 μm s^−1^. Helium (600 ml/min) carried the ablated sample from the sample cell, before mixing with nitrogen (5 ml/min) and the instrument nebuliser flow (argon at 0.85 L/min) to pass through a smoothing device "The SQUID" before reaching the plasma torch. Mass spectrometry was conducted with a dwell time of 50 ms. All elements were referenced to NIST SRM612. As the NIST glass references are not certified for Mg, the reference value supplied by the Iolite software (77 ppm) was used^61^. Mass spectrometry data were processed with Iolite normalised to ^43^Ca as the Internal Standard.

### Age model

The age model was constructed using an ensemble of 30 iterations of the StalAge algorithm, with 8 U/Th dates which spanned an apparent hiatus in the speleothem’s growth (Supplementary information). While age models for stalagmites with known hiatuses can be calculated in discrete sections^62^, StalAge is capable of successfully modelling hiatuses and their increased uncertainty^24,62,63^.

Using StalAge we modelled independently the ages of the two sections either side of the apparent growth hiatus in GC001 as well as one continuous age model using an ensemble of 30 iterations each. The maximum difference between the two discrete age models and the continuous age model for GC001 was just 30 years, well within the 2σ error of the ages. As such, we use the one age model over the whole stalagmite. Points between 100 and 140 mm were excluded to ensure the growth hiatus was avoided. Full uranium-series dating results are presented in Table S1. None of the data post growth hiatus are presented here since this research focused on MIS 5e.

### Analysis of geochemical time series

Mineral weathering of the local karst is the dominant source of Mg and Sr in speleothems in the Yarrangobilly caves^27^, with drip-water monitoring showing that Mg and Sr (and Ca) can be used as paleo-environmental proxies for contrasting rainfall conditions^26,27^. Namely, reduced rainfall invokes prior calcite precipitation (PCP) and increased drip-water concentrations, and hence stalagmite Mg/Ca (and at times Sr/Ca) ratios^43^. As drip-water monitoring before GC001 was collected was not possible we calculated the slope of ln(Sr/Ca) vs ln(Mg/Ca) (n = 6000 pairs of ten point running means). A positive correlation with a slope of 0.88 was calculated confirming Mg and Sr variations in GC001 are under some form of hydrological control^64^.

Extreme outliers (> 3 σ) in the time series of ^25^ Mg, ^88^Sr and ^137^Ba data were removed with the gaps filled using a kalman filter. Three periods of missing data due to saw cuts at 118,292 years BP lasting for 82 years, at 120,104 years BP lasting for 64 years and, at 122,099 years BP lasting for 80 years were filled also using a kalman filter. Lomb-Scargle periodograms, which are commonly used in frequency analysis of unequally spaced data including paleo-environmental data, were then developed using the raw filtered data. The data was then resampled at 1 year time steps, using linear interpolation, prior to calculating the wavelet transforms.

## Supplementary information

Supplementary Information.

## Data Availability

All data used in this research is archived and accessible through The University of Queensland eSpace Library or by contacting HM.
