# Supplementary material for: Evidence of wet-dry cycles and mega-droughts in the Eemian climate of southeast Australia
Source: Sci Rep. 2020 Oct 22;10:18000. doi: 10.1038/s41598-020-75071-z (PMC7581751; doi:10.1038/s41598-020-75071-z)
Supplement: Supplementary file 1 — Supplementary Information. [file 41598_2020_75071_MOESM1_ESM.docx]

**Supplementary Information for:**

Evidence of wet-dry cycles and mega-droughts in the Eemian climate of southeast Australia.

**Authors:**

^1#^Hamish McGowan, ^2^Micheline Campbell, ^2^John Nikolaus Callow, ^1^Andrew Lowry and ^3^Henri Wong

^1^Atmospheric Observations Research Group, The University of Queensland, Brisbane, Australia.

^2^School of Agriculture and Environment, The University of Western Australia, Perth, Australia.

^3^Australian Nuclear Science and Technology Organisation, Lucas Heights, Sydney, Australia.

**Introduction**

This supporting information provides:

Table S1 presents details of the eight uranium series dates obtained for stalagmite GC001. The age model was developed using the StalAge algorithm of *Scholz and Hoffmann,* [2011]. Ensemble means for the 8 dates and their ± 2δ output from the StalAge algorithm are presented in Table S2 and shown graphically in Figure S1.

**Table S1**. Uranium series dating results for 8 sub-samples milled from stalagmite GC001 with ages shown in years BP (1950) with ± 2σ, and respective year BCE.

Note: Ratios listed in the table refer to activity ratios normalized to the corresponding ratios measured for the secular-equilibrium HU-1 standard. ^230^Th ages are calculated using Isoplot/Ex 3.75 [*Ludwig,* 2012], using decay constants of Cheng et al., [2000]. Non-radiogenic ^230^Th correction was applied assuming non-radiogenic 230Th/232Th atomic ratio = 4.4±2.2 x 10^-6^ (bulk-earth value), and ^238^U, ^234^U, ^232^Th and ^230^Th are in secular equilibrium. Non-radiogenic ^230^Th correction results in large age error magnification for samples with low ^230^Th/^232^Th ratios. uncorr. and corr. denote uncorrected and corrected.

**Table S2**. Presented ages (rounded to nearest year) are the original corrected ^230^Th age in years (BP) and mean ages from a 30 iteration ensemble from StalAge. Standard 2δ are also presented.

| **Depth from tip of GC001 (mm)** | **Original corrected ^230^Th Age years (BP)** | **+/- 2δ years** | **Corrected StalAge mean ^230^Th Age years (BP) (n=30)** |
| --- | --- | --- | --- |
| 23 | 103289 | 484 | 103113 |
| 60 | 104030 | 818 | 104601 |
| 100 | 106521 | 897 | 106191 |
| 140 | 118254 | 927 | 117968 |
| 178 | 119115 | 526 | 119285 |
| 220 | 120859 | 845 | 120933 |
| 260 | 123312 | 1593 | 122476 |
| 292 | 123251 | 709 | 123514 |


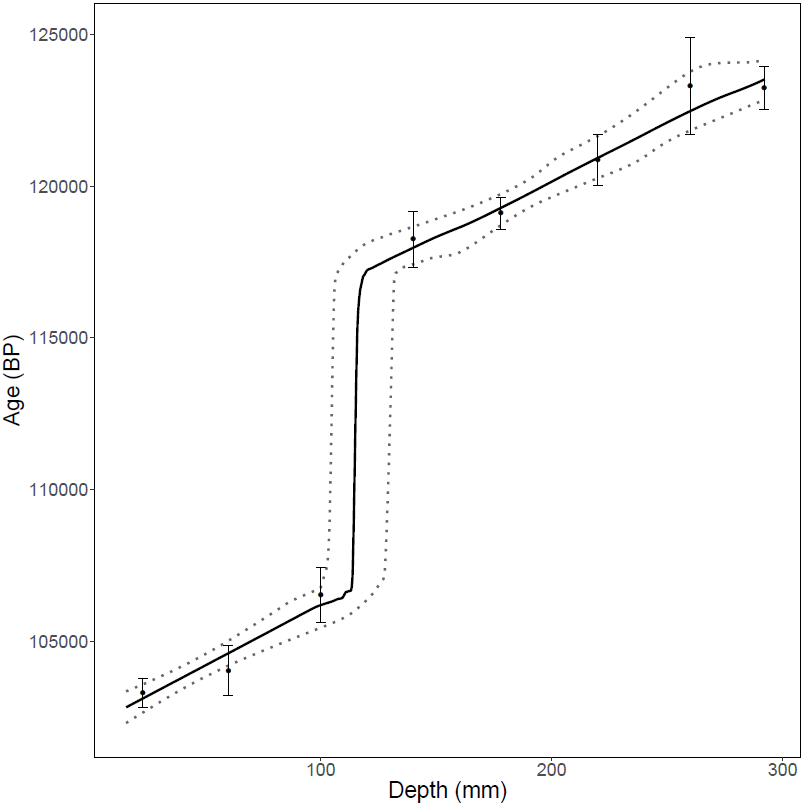


**Figure S1.** Final age model (solid black line) for GC001 developed using the StalAge algorithm [Scholz and Hoffmann, 2011] for the entire length of the stalagmite with 95%-confidence limits shown as dashed lines.
